# Biomechanics data of human supra-aortic trunks and abdominal visceral arteries harvested during autopsy

**DOI:** 10.1016/j.dib.2020.106569

**Published:** 2020-11-23

**Authors:** Vivian Carla Gomes, Jorge Gomes, Gina Camillo Silvestre, Alexandre Queiroz, Michele Alberto Marques, Erasmo Simão da Silva

**Affiliations:** a Vascular and Endovascular Surgery Division, Department of Surgery, Laboratory for Medical Investigation #2, (LIM 02). University of São Paulo School of Medicine (FMUSP). Dr. Arnaldo, 455 – room 1312 - Cerqueira César, Postal code: 01246-903 - São Paulo - SP, Brasil; b Shamah Engineering,. Fagundes Filho, 361, room 11, São Judas, Postal code: 04304-010, São Paulo, SP, Brasil

**Keywords:** Biomechanics, Uniaxial tensile test, Stress, Supra-aortic trunks, Renal arteries, Superior mesenteric artery, Celiac trunk, Autopsy

## Abstract

The present dataset describes the biomechanical properties of the supra-aortic trunks (brachiocephalic trunk, left common carotid artery, and left subclavian artery) and some of the visceral branches of the abdominal aorta (celiac trunk, superior mesenteric artery, and renal arteries). The specimens have been harvested from 27 adult donors during the autopsy procedure. The vessels were submitted to uniaxial biomechanical tensile tests, and values of failure stress, failure tension, and failure strain were obtained. As atherosclerosis could affect any of those vessels producing a significant reduction in their lumen, the data presented here could be of great interest to vascular surgeons, interventional cardiologists, and interventional neuroradiologists, who manipulate these arteries endovascularly. The observations gathered here are experimental evidence of the vessels’ endurance against tearing and of their deformability. Therefore this data article could also help the medical industry dedicated to the production of endovascular devices. This dataset is related to the article entitled “Left Common Carotid Artery Biomechanical Properties in Individuals over 80 years: Women Have Stiffer Vessels” published in Annals of Vascular Surgery in August 2020 [1].

## Specifications Table

**Table:** 

Subject	Cardiovascular Medicine
Specific subject area	Vascular surgery; Vascular biomechanics
Type of data	Table Graph
How data were acquired	- Biomechanic uniaxial tensile test - Instruments: Biomechanic tensile test equipment - Make and model of the instruments used: * INSTRON SPEC 2200 / INSPEC software
Data format	Raw
Parameters for data collection	The cadaveric specimens were randomly harvested.
Description of data collection	During autopsy procedure, the whole aorta (thoracic and abdominal parts) was harvested along with its main branches (brachiocephalic trunk, left common carotid artery, left subclavian artery, celiac trunk, superior mesenteric artery and renal arteries). These branches were carefully dissected and collected for biomechanical analysis through uniaxial tensile test, which evaluated the following variables: failure stress, failure tension, and failure strain.
Data source location	**Institution:** University of São Paulo School of Medicine **City/Town/Region:** São Paulo, São Paulo, Southeast region **Country:** Brazil **Latitude and longitude for collected samples/data:** 23°33′20.7″S 46°40′13.0″W **Primary data sources:** **Institution:** University of São Paulo School of Medicine **City/Town/Region:** São Paulo, São Paulo, Southeast region **Country:** Brazil **Latitude and longitude for collected samples/data:** 23°33′20.7″S 46°40′13.0″W
Data accessibility	**Repository name:** Dataset on biomechanics of human supra-aortic trunks and abdominal visceral arteries harvested during autopsy [2] **Data identification number:** 10.17632/v9v6ck223r.1 **Direct URL to data:**https://doi.org/10.17632/v9v6ck223r.1
Related research article	V.C. Gomes, L.F. da Silva, S.P. Zyngier, et al., Left Common Carotid Artery Biomechanical Properties in Individuals over 80 years: Women Have Stiffer Vessels, Ann. Vasc. Surg. 67 (2020) 461–467.1 https://doi.org/10.1016/j.avsg.2020.01.107

## Value of the Data

• The present data are experimental information regarding the biomechanical behavior of supra-aortic trunks and visceral arteries. Considering that some of those arteries are frequently manipulated during endovascular procedures, it is of great relevance knowing their resistance against tearing and compliance.
• As these arteries are manipulated during endovascular procedures, their biomechanical behavior could be precious information, especially to vascular surgeons, interventional neuroradiologists as well as to interventional cardiologists.
• The present data could be especially useful for the development of new endovascular materials, like stents and catheters, used in the supra-aortic trunks and visceral arteries.
• The knowledge of the biomechanical properties of the abovementioned arteries could better instruct the professionals and industry that deal with this vessels, aiming safer surgical procedures and high quality endovascular devices.

## 1 Data Description

**Table:** 

File name	File Description
CASE 1	

CASE1aret Curve Fitted.jpg	Biomechanical test of left renal artery – chart represents the elastic diagram related to this uniaxial tensile test. The elastic diagram (stress-strain curve) is built with force and elongation information until the sample rupture.
CASE1aret Specimen 1 Data File.xls	Biomechanical test of left renal artery - excel file related to the chart: this excel file contains precisely all the information that enabled the creation of the chart related to this sample. It comprises critical data such as displacement observed during the tensile test (mm), the load applied (N), strain (deformation), tension (N/cm), and stress (N/m2).
CASE1aret.TXT	Biomechanical test of left renal artery - biomechanical test report: the biomechanical test device produces a report detailing the experiments' conditions, such as temperature and humidity, as well as the width and thickness of the sample.
CASE 2	

CASE2accet Curve Fitted.jpg	Biomechanical test of left common carotid artery – chart represents the elastic diagram related to this uniaxial tensile test. The elastic diagram (stress-strain curve) is built with force and elongation information until the sample rupture.
CASE2accet Specimen 1 Data File.xls	Biomechanical test of left common carotid artery - excel file related to the chart: this excel file contains precisely all the information that enabled the creation of the chart related to this sample. It comprises critical data such as displacement observed during the tensile test (mm), the load applied (N), strain (deformation), tension (N/cm), and stress (N/m2).
CASE2accet.TXT	Biomechanical test of left common carotid artery - biomechanical test report: the biomechanical test device produces a report detailing the experiments' conditions, such as temperature and humidity, as well as the width and thickness of the sample.
CASE2aret Curve Fitted.jpg	Biomechanical test of left renal artery – chart represents the elastic diagram related to this uniaxial tensile test. The elastic diagram (stress-strain curve) is built with force and elongation information until the sample rupture.
CASE2aret Specimen 1 Data File.xls	Biomechanical test of left renal artery - excel file related to the chart: this excel file contains precisely all the information that enabled the creation of the chart related to this sample. It comprises critical data such as displacement observed during the tensile test (mm), the load applied (N), strain (deformation), tension (N/cm), and stress (N/m2).
CASE2aret.TXT	Biomechanical test of left renal artery - biomechanical test report: the biomechanical test device produces a report detailing the experiments' conditions, such as temperature and humidity, as well as the width and thickness of the sample.
CASE2ascet Curve Fitted.jpg	Biomechanical test of left subclavian artery – chart represents the elastic diagram related to this uniaxial tensile test. The elastic diagram (stress-strain curve) is built with force and elongation information until the sample rupture.
CASE2ascet Specimen 1 Data File.xls	Biomechanical test of left subclavian artery - excel file related to the chart: this excel file contains precisely all the information that enabled the creation of the chart related to this sample. It comprises critical data such as displacement observed during the tensile test (mm), the load applied (N), strain (deformation), tension (N/cm), and stress (N/m2).
CASE2ascet.TXT	Biomechanical test of left subclavian artery - biomechanical test report: the biomechanical test device produces a report detailing the experiments' conditions, such as temperature and humidity, as well as the width and thickness of the sample.
CASE2tbct Curve Fitted.jpg	Biomechanical test of brachiocephalic trunk – chart represents the elastic diagram related to this uniaxial tensile test. The elastic diagram (stress-strain curve) is built with force and elongation information until the sample rupture.
CASE2tbct Specimen 1 Data File.xls	Biomechanical test of brachiocephalic trunk - excel file related to the chart: this excel file contains precisely all the information that enabled the creation of the chart related to this sample. It comprises critical data such as displacement observed during the tensile test (mm), the load applied (N), strain (deformation), tension (N/cm), and stress (N/m2).
CASE2tbct.TXT	Biomechanical test of brachiocephalic trunk – biomechanical test report: the biomechanical test device produces a report detailing the experiments' conditions, such as temperature and humidity, as well as the width and thickness of the sample.
CASE 3	

CASE3accet Curve Fitted.jpg	Biomechanical test of left common carotid artery – chart represents the elastic diagram related to this uniaxial tensile test. The elastic diagram (stress-strain curve) is built with force and elongation information until the sample rupture.
CASE3accet Specimen 1 Data File.xls	Biomechanical test of left common carotid artery - excel file related to the chart: this excel file contains precisely all the information that enabled the creation of the chart related to this sample. It comprises critical data such as displacement observed during the tensile test (mm), the load applied (N), strain (deformation), tension (N/cm), and stress (N/m2).
CASE3accet.TXT	Biomechanical test of left common carotid artery - biomechanical test report: the biomechanical test device produces a report detailing the experiments' conditions, such as temperature and humidity, as well as the width and thickness of the sample.
CASE3amst Curve Fitted.jpg	Biomechanical test of superior mesenteric artery – chart represents the elastic diagram related to this uniaxial tensile test. The elastic diagram (stress-strain curve) is built with force and elongation information until the sample rupture.
CASE3amst Specimen 1 Data File.xls	Biomechanical test of superior mesenteric artery - excel file related to the chart: this excel file contains precisely all the information that enabled the creation of the chart related to this sample. It comprises critical data such as displacement observed during the tensile test (mm), the load applied (N), strain (deformation), tension (N/cm), and stress (N/m2).
CASE3amst.TXT	Biomechanical test of superior mesenteric artery - biomechanical test report: the biomechanical test device produces a report detailing the experiments' conditions, such as temperature and humidity, as well as the width and thickness of the sample.
CASE3aret Curve Fitted.jpg	Biomechanical test of left renal artery – chart represents the elastic diagram related to this uniaxial tensile test. The elastic diagram (stress-strain curve) is built with force and elongation information until the sample rupture.
CASE3aret Specimen 1 Data File.xls	Biomechanical test of left renal artery - excel file related to the chart: this excel file contains precisely all the information that enabled the creation of the chart related to this sample. It comprises critical data such as displacement observed during the tensile test (mm), the load applied (N), strain (deformation), tension (N/cm), and stress (N/m2).
CASE3aret.TXT	Biomechanical test of left renal artery - biomechanical test report: the biomechanical test device produces a report detailing the experiments' conditions, such as temperature and humidity, as well as the width and thickness of the sample.
CASE3ascet Curve Fitted.jpg	Biomechanical test of left subclavian artery – chart represents the elastic diagram related to this uniaxial tensile test. The elastic diagram (stress-strain curve) is built with force and elongation information until the sample rupture.
CASE3ascet Specimen 1 Data File.xls	Biomechanical test of left subclavian artery - excel file related to the chart: this excel file contains precisely all the information that enabled the creation of the chart related to this sample. It comprises critical data such as displacement observed during the tensile test (mm), the load applied (N), strain (deformation), tension (N/cm), and stress (N/m2).
CASE3ascet.TXT	Biomechanical test of left subclavian artery - biomechanical test report: the biomechanical test device produces a report detailing the experiments' conditions, such as temperature and humidity, as well as the width and thickness of the sample.
CASE3tbct Curve Fitted.jpg	Biomechanical test of brachiocephalic trunk – chart represents the elastic diagram related to this uniaxial tensile test. The elastic diagram (stress-strain curve) is built with force and elongation information until the sample rupture.
CASE3tbct Specimen 1 Data File.xls	Biomechanical test of brachiocephalic trunk - excel file related to the chart: this excel file contains precisely all the information that enabled the creation of the chart related to this sample. It comprises critical data such as displacement observed during the tensile test (mm), the load applied (N), strain (deformation), tension (N/cm), and stress (N/m2).
CASE3tbct.TXT	Biomechanical test of brachiocephalic trunk – biomechanical test report: the biomechanical test device produces a report detailing the experiments' conditions, such as temperature and humidity, as well as the width and thickness of the sample.
CASE 4	

CASE4aret Curve Fitted.jpg	Biomechanical test of left renal artery – chart represents the elastic diagram related to this uniaxial tensile test. The elastic diagram (stress-strain curve) is built with force and elongation information until the sample rupture.
CASE4aret Specimen 1 Data File.xls	Biomechanical test of left renal artery - excel file related to the chart: this excel file contains precisely all the information that enabled the creation of the chart related to this sample. It comprises critical data such as displacement observed during the tensile test (mm), the load applied (N), strain (deformation), tension (N/cm), and stress (N/m2).
CASE4aret.TXT	Biomechanical test of left renal artery - biomechanical test report: the biomechanical test device produces a report detailing the experiments' conditions, such as temperature and humidity, as well as the width and thickness of the sample.
CASE 5	

CASE5tct Curve Fitted.jpg	Biomechanical test of celiac trunk – chart represents the elastic diagram related to this uniaxial tensile test. The elastic diagram (stress-strain curve) is built with force and elongation information until the sample rupture.
CASE5tct Specimen 1 Data File.xls	Biomechanical test of celiac trunk - excel file related to the chart: this excel file contains precisely all the information that enabled the creation of the chart related to this sample. It comprises critical data such as displacement observed during the tensile test (mm), the load applied (N), strain (deformation), tension (N/cm), and stress (N/m2).
CASE5tct.TXT	Biomechanical test of celiac trunk - biomechanical test report: the biomechanical test device produces a report detailing the experiments' conditions, such as temperature and humidity, as well as the width and thickness of the sample.
CASE 6	

CASE6accet Curve Fitted.jpg	Biomechanical test of left common carotid artery – chart represents the elastic diagram related to this uniaxial tensile test. The elastic diagram (stress-strain curve) is built with force and elongation information until the sample rupture.
CASE6accet Specimen 1 Data File.xls	Biomechanical test of left common carotid artery - excel file related to the chart: this excel file contains precisely all the information that enabled the creation of the chart related to this sample. It comprises critical data such as displacement observed during the tensile test (mm), the load applied (N), strain (deformation), tension (N/cm), and stress (N/m2).
CASE6accet.TXT	Biomechanical test of left common carotid artery - biomechanical test report: the biomechanical test device produces a report detailing the experiments' conditions, such as temperature and humidity, as well as the width and thickness of the sample.
CASE6aret Curve Fitted.jpg	Biomechanical test of left renal artery – chart represents the elastic diagram related to this uniaxial tensile test. The elastic diagram (stress-strain curve) is built with force and elongation information until the sample rupture.
CASE6aret Specimen 1 Data File.xls	Biomechanical test of left renal artery - excel file related to the chart: this excel file contains precisely all the information that enabled the creation of the chart related to this sample. It comprises critical data such as displacement observed during the tensile test (mm), the load applied (N), strain (deformation), tension (N/cm), and stress (N/m2).
CASE6aret.TXT	Biomechanical test of left renal artery - biomechanical test report: the biomechanical test device produces a report detailing the experiments' conditions, such as temperature and humidity, as well as the width and thickness of the sample.
CASE6ascet Curve Fitted.jpg	Biomechanical test of left subclavian artery – chart represents the elastic diagram related to this uniaxial tensile test. The elastic diagram (stress-strain curve) is built with force and elongation information until the sample rupture.
CASE6ascet Specimen 1 Data File.xls	Biomechanical test of left subclavian artery - excel file related to the chart: this excel file contains precisely all the information that enabled the creation of the chart related to this sample. It comprises critical data such as displacement observed during the tensile test (mm), the load applied (N), strain (deformation), tension (N/cm), and stress (N/m2).
CASE6ascet.TXT	Biomechanical test of left subclavian artery - biomechanical test report: the biomechanical test device produces a report detailing the experiments' conditions, such as temperature and humidity, as well as the width and thickness of the sample.
CASE6tbct Curve Fitted.jpg	Biomechanical test of brachiocephalic trunk – chart represents the elastic diagram related to this uniaxial tensile test. The elastic diagram (stress-strain curve) is built with force and elongation information until the sample rupture.
CASE6tbct Specimen 1 Data File.xls	Biomechanical test of brachiocephalic trunk - excel file related to the chart: this excel file contains precisely all the information that enabled the creation of the chart related to this sample. It comprises critical data such as displacement observed during the tensile test (mm), the load applied (N), strain (deformation), tension (N/cm), and stress (N/m2).
CASE6tbct.TXT	Biomechanical test of brachiocephalic trunk – biomechanical test report: the biomechanical test device produces a report detailing the experiments' conditions, such as temperature and humidity, as well as the width and thickness of the sample.
CASE 7	

CASE6accet Curve Fitted.jpg	Biomechanical test of left common carotid artery – chart represents the elastic diagram related to this uniaxial tensile test. The elastic diagram (stress-strain curve) is built with force and elongation information until the sample rupture.
CASE6accet Specimen 1 Data File.xls	Biomechanical test of left common carotid artery - excel file related to the chart: this excel file contains precisely all the information that enabled the creation of the chart related to this sample. It comprises critical data such as displacement observed during the tensile test (mm), the load applied (N), strain (deformation), tension (N/cm), and stress (N/m2).
CASE6accet.TXT	Biomechanical test of left common carotid artery - biomechanical test report: the biomechanical test device produces a report detailing the experiments' conditions, such as temperature and humidity, as well as the width and thickness of the sample.
CASE6aret Curve Fitted.jpg	Biomechanical test of left renal artery – chart represents the elastic diagram related to this uniaxial tensile test. The elastic diagram (stress-strain curve) is built with force and elongation information until the sample rupture.
CASE6aret Specimen 1 Data File.xls	Biomechanical test of left renal artery - excel file related to the chart: this excel file contains precisely all the information that enabled the creation of the chart related to this sample. It comprises critical data such as displacement observed during the tensile test (mm), the load applied (N), strain (deformation), tension (N/cm), and stress (N/m2).
CASE6aret.TXT	Biomechanical test of left renal artery - biomechanical test report: the biomechanical test device produces a report detailing the experiments' conditions, such as temperature and humidity, as well as the width and thickness of the sample.
CASE6ascet Curve Fitted.jpg	Biomechanical test of left subclavian artery – chart represents the elastic diagram related to this uniaxial tensile test. The elastic diagram (stress-strain curve) is built with force and elongation information until the sample rupture.
CASE6ascet Specimen 1 Data File.xls	Biomechanical test of left subclavian artery - excel file related to the chart: this excel file contains precisely all the information that enabled the creation of the chart related to this sample. It comprises critical data such as displacement observed during the tensile test (mm), the load applied (N), strain (deformation), tension (N/cm), and stress (N/m2).
CASE6ascet.TXT	Biomechanical test of left subclavian artery - biomechanical test report: the biomechanical test device produces a report detailing the experiments' conditions, such as temperature and humidity, as well as the width and thickness of the sample.
CASE6tbct Curve Fitted.jpg	Biomechanical test of brachiocephalic trunk – chart represents the elastic diagram related to this uniaxial tensile test. The elastic diagram (stress-strain curve) is built with force and elongation information until the sample rupture.
CASE6tbct Specimen 1 Data File.xls	Biomechanical test of brachiocephalic trunk - excel file related to the chart: this excel file contains precisely all the information that enabled the creation of the chart related to this sample. It comprises critical data such as displacement observed during the tensile test (mm), the load applied (N), strain (deformation), tension (N/cm), and stress (N/m2).
CASE6tbct.TXT	Biomechanical test of brachiocephalic trunk – biomechanical test report: the biomechanical test device produces a report detailing the experiments' conditions, such as temperature and humidity, as well as the width and thickness of the sample.
CASE 8	

CASE8accet Curve Fitted.jpg	Biomechanical test of left common carotid artery – chart represents the elastic diagram related to this uniaxial tensile test. The elastic diagram (stress-strain curve) is built with force and elongation information until the sample rupture.
CASE8accet Specimen 1 Data File.xls	Biomechanical test of left common carotid artery - excel file related to the chart: this excel file contains precisely all the information that enabled the creation of the chart related to this sample. It comprises critical data such as displacement observed during the tensile test (mm), the load applied (N), strain (deformation), tension (N/cm), and stress (N/m2).
CASE8accet.TXT	Biomechanical test of left common carotid artery - biomechanical test report: the biomechanical test device produces a report detailing the experiments' conditions, such as temperature and humidity, as well as the width and thickness of the sample.
CASE8ascet Curve Fitted.jpg	Biomechanical test of left subclavian artery – chart represents the elastic diagram related to this uniaxial tensile test. The elastic diagram (stress-strain curve) is built with force and elongation information until the sample rupture.
CASE8ascet Specimen 1 Data File.xls	Biomechanical test of left subclavian artery - excel file related to the chart: this excel file contains precisely all the information that enabled the creation of the chart related to this sample. It comprises critical data such as displacement observed during the tensile test (mm), the load applied (N), strain (deformation), tension (N/cm), and stress (N/m2).
CASE8ascet.TXT	Biomechanical test of left subclavian artery - biomechanical test report: the biomechanical test device produces a report detailing the experiments' conditions, such as temperature and humidity, as well as the width and thickness of the sample.
CASE8tbct Curve Fitted.jpg	Biomechanical test of brachiocephalic trunk – chart represents the elastic diagram related to this uniaxial tensile test. The elastic diagram (stress-strain curve) is built with force and elongation information until the sample rupture.
CASE8tbct Specimen 1 Data File.xls	Biomechanical test of brachiocephalic trunk - excel file related to the chart: this excel file contains precisely all the information that enabled the creation of the chart related to this sample. It comprises critical data such as displacement observed during the tensile test (mm), the load applied (N), strain (deformation), tension (N/cm), and stress (N/m2).
CASE8tbct.TXT	Biomechanical test of brachiocephalic trunk – biomechanical test report: the biomechanical test device produces a report detailing the experiments' conditions, such as temperature and humidity, as well as the width and thickness of the sample.
CASE 9	

CASE9amst Curve Fitted.jpg	Biomechanical test of superior mesenteric artery – chart represents the elastic diagram related to this uniaxial tensile test. The elastic diagram (stress-strain curve) is built with force and elongation information until the sample rupture.
CASE9amst Specimen 1 Data File.xls	Biomechanical test of superior mesenteric artery - excel file related to the chart: this excel file contains precisely all the information that enabled the creation of the chart related to this sample. It comprises critical data such as displacement observed during the tensile test (mm), the load applied (N), strain (deformation), tension (N/cm), and stress (N/m2).
CASE9amst.TXT	Biomechanical test of superior mesenteric artery - biomechanical test report: the biomechanical test device produces a report detailing the experiments' conditions, such as temperature and humidity, as well as the width and thickness of the sample.
CASE9ardt Curve Fitted.jpg	Biomechanical test of right renal artery – chart represents the elastic diagram related to this uniaxial tensile test. The elastic diagram (stress-strain curve) is built with force and elongation information until the sample rupture.
CASE9ardt Specimen 1 Data File.xls	Biomechanical test of right renal artery - excel file related to the chart: this excel file contains precisely all the information that enabled the creation of the chart related to this sample. It comprises critical data such as displacement observed during the tensile test (mm), the load applied (N), strain (deformation), tension (N/cm), and stress (N/m2).
CASE9ardt.TXT	Biomechanical test of right renal artery - biomechanical test report: the biomechanical test device produces a report detailing the experiments' conditions, such as temperature and humidity, as well as the width and thickness of the sample.
CASE9ascet Curve Fitted.jpg	Biomechanical test of left subclavian artery – chart represents the elastic diagram related to this uniaxial tensile test. The elastic diagram (stress-strain curve) is built with force and elongation information until the sample rupture.
CASE9ascet Specimen 1 Data File.xls	Biomechanical test of left subclavian artery - excel file related to the chart: this excel file contains precisely all the information that enabled the creation of the chart related to this sample. It comprises critical data such as displacement observed during the tensile test (mm), the load applied (N), strain (deformation), tension (N/cm), and stress (N/m2).
CASE9ascet.TXT	Biomechanical test of left subclavian artery - biomechanical test report: the biomechanical test device produces a report detailing the experiments' conditions, such as temperature and humidity, as well as the width and thickness of the sample.
CASE9tbct Curve Fitted.jpg	Biomechanical test of brachiocephalic trunk – chart represents the elastic diagram related to this uniaxial tensile test. The elastic diagram (stress-strain curve) is built with force and elongation information until the sample rupture.
CASE9tbct Specimen 1 Data File.xls	Biomechanical test of brachiocephalic trunk - excel file related to the chart: this excel file contains precisely all the information that enabled the creation of the chart related to this sample. It comprises critical data such as displacement observed during the tensile test (mm), the load applied (N), strain (deformation), tension (N/cm), and stress (N/m2).
CASE9tbct.TXT	Biomechanical test of brachiocephalic trunk – biomechanical test report: the biomechanical test device produces a report detailing the experiments' conditions, such as temperature and humidity, as well as the width and thickness of the sample.
CASE9tct Curve Fitted.jpg	Biomechanical test of celiac trunk – chart represents the elastic diagram related to this uniaxial tensile test. The elastic diagram (stress-strain curve) is built with force and elongation information until the sample rupture.
CASE9tct Specimen 1 Data File.xls	Biomechanical test of celiac trunk - excel file related to the chart: this excel file contains precisely all the information that enabled the creation of the chart related to this sample. It comprises critical data such as displacement observed during the tensile test (mm), the load applied (N), strain (deformation), tension (N/cm), and stress (N/m2).
CASE9tct.TXT	Biomechanical test of celiac trunk - biomechanical test report: the biomechanical test device produces a report detailing the experiments' conditions, such as temperature and humidity, as well as the width and thickness of the sample.
CASE 10	

CASE10accet Curve Fitted.jpg	Biomechanical test of left common carotid artery – chart represents the elastic diagram related to this uniaxial tensile test. The elastic diagram (stress-strain curve) is built with force and elongation information until the sample rupture.
CASE10accet Specimen 1 Data File.xls	Biomechanical test of left common carotid artery - excel file related to the chart: this excel file contains precisely all the information that enabled the creation of the chart related to this sample. It comprises critical data such as displacement observed during the tensile test (mm), the load applied (N), strain (deformation), tension (N/cm), and stress (N/m2).
CASE10accet.TXT	Biomechanical test of left common carotid artery - biomechanical test report: the biomechanical test device produces a report detailing the experiments' conditions, such as temperature and humidity, as well as the width and thickness of the sample.
CASE10amst Curve Fitted.jpg	Biomechanical test of superior mesenteric artery – chart represents the elastic diagram related to this uniaxial tensile test. The elastic diagram (stress-strain curve) is built with force and elongation information until the sample rupture.
CASE10amst Specimen 1 Data File.xls	Biomechanical test of superior mesenteric artery - excel file related to the chart: this excel file contains precisely all the information that enabled the creation of the chart related to this sample. It comprises critical data such as displacement observed during the tensile test (mm), the load applied (N), strain (deformation), tension (N/cm), and stress (N/m2).
CASE10amst.TXT	Biomechanical test of superior mesenteric artery - biomechanical test report: the biomechanical test device produces a report detailing the experiments' conditions, such as temperature and humidity, as well as the width and thickness of the sample.
CASE10ardt Curve Fitted.jpg	Biomechanical test of right renal artery – chart represents the elastic diagram related to this uniaxial tensile test. The elastic diagram (stress-strain curve) is built with force and elongation information until the sample rupture.
CASE10ardt Specimen 1 Data File.xls	Biomechanical test of right renal artery - excel file related to the chart: this excel file contains precisely all the information that enabled the creation of the chart related to this sample. It comprises critical data such as displacement observed during the tensile test (mm), the load applied (N), strain (deformation), tension (N/cm), and stress (N/m2).
CASE10ardt.TXT	Biomechanical test of right renal artery - biomechanical test report: the biomechanical test device produces a report detailing the experiments' conditions, such as temperature and humidity, as well as the width and thickness of the sample.
CASE10ascet Curve Fitted.jpg	Biomechanical test of left subclavian artery – chart represents the elastic diagram related to this uniaxial tensile test. The elastic diagram (stress-strain curve) is built with force and elongation information until the sample rupture.
CASE10ascet Specimen 1 Data File.xls	Biomechanical test of left subclavian artery - excel file related to the chart: this excel file contains precisely all the information that enabled the creation of the chart related to this sample. It comprises critical data such as displacement observed during the tensile test (mm), the load applied (N), strain (deformation), tension (N/cm), and stress (N/m2).
CASE10ascet.TXT	Biomechanical test of left subclavian artery - biomechanical test report: the biomechanical test device produces a report detailing the experiments' conditions, such as temperature and humidity, as well as the width and thickness of the sample.
CASE10tbct Curve Fitted.jpg	Biomechanical test of brachiocephalic trunk – chart represents the elastic diagram related to this uniaxial tensile test. The elastic diagram (stress-strain curve) is built with force and elongation information until the sample rupture.
CASE10tbct Specimen 1 Data File.xls	Biomechanical test of brachiocephalic trunk - excel file related to the chart: this excel file contains precisely all the information that enabled the creation of the chart related to this sample. It comprises critical data such as displacement observed during the tensile test (mm), the load applied (N), strain (deformation), tension (N/cm), and stress (N/m2).
CASE10tbct.TXT	Biomechanical test of brachiocephalic trunk – biomechanical test report: the biomechanical test device produces a report detailing the experiments' conditions, such as temperature and humidity, as well as the width and thickness of the sample.
CASE10tct Curve Fitted.jpg	Biomechanical test of celiac trunk – chart represents the elastic diagram related to this uniaxial tensile test. The elastic diagram (stress-strain curve) is built with force and elongation information until the sample rupture.
CASE10tct Specimen 1 Data File.xls	Biomechanical test of celiac trunk - excel file related to the chart: this excel file contains precisely all the information that enabled the creation of the chart related to this sample. It comprises critical data such as displacement observed during the tensile test (mm), the load applied (N), strain (deformation), tension (N/cm), and stress (N/m2).
CASE10tct.TXT	Biomechanical test of celiac trunk - biomechanical test report: the biomechanical test device produces a report detailing the experiments' conditions, such as temperature and humidity, as well as the width and thickness of the sample.
CASE 11	

CASE11accet Curve Fitted.jpg	Biomechanical test of left common carotid artery – chart represents the elastic diagram related to this uniaxial tensile test. The elastic diagram (stress-strain curve) is built with force and elongation information until the sample rupture.
CASE11accet Specimen 1 Data File.xls	Biomechanical test of left common carotid artery - excel file related to the chart: this excel file contains precisely all the information that enabled the creation of the chart related to this sample. It comprises critical data such as displacement observed during the tensile test (mm), the load applied (N), strain (deformation), tension (N/cm), and stress (N/m2).
CASE11accet.TXT	Biomechanical test of left common carotid artery - biomechanical test report: the biomechanical test device produces a report detailing the experiments' conditions, such as temperature and humidity, as well as the width and thickness of the sample.
CASE11ascet Curve Fitted.jpg	Biomechanical test of left subclavian artery – chart represents the elastic diagram related to this uniaxial tensile test. The elastic diagram (stress-strain curve) is built with force and elongation information until the sample rupture.
CASE11ascet Specimen 1 Data File.xls	Biomechanical test of left subclavian artery - excel file related to the chart: this excel file contains precisely all the information that enabled the creation of the chart related to this sample. It comprises critical data such as displacement observed during the tensile test (mm), the load applied (N), strain (deformation), tension (N/cm), and stress (N/m2).
CASE11ascet.TXT	Biomechanical test of left subclavian artery - biomechanical test report: the biomechanical test device produces a report detailing the experiments' conditions, such as temperature and humidity, as well as the width and thickness of the sample.
CASE11tbct Curve Fitted.jpg	Biomechanical test of brachiocephalic trunk – chart represents the elastic diagram related to this uniaxial tensile test. The elastic diagram (stress-strain curve) is built with force and elongation information until the sample rupture.
CASE11tbct Specimen 1 Data File.xls	Biomechanical test of brachiocephalic trunk - excel file related to the chart: this excel file contains precisely all the information that enabled the creation of the chart related to this sample. It comprises critical data such as displacement observed during the tensile test (mm), the load applied (N), strain (deformation), tension (N/cm), and stress (N/m2).
CASE11tbct.TXT	Biomechanical test of brachiocephalic trunk – biomechanical test report: the biomechanical test device produces a report detailing the experiments' conditions, such as temperature and humidity, as well as the width and thickness of the sample.
CASE 12	

CASE12accet Curve Fitted.jpg	Biomechanical test of left common carotid artery – chart represents the elastic diagram related to this uniaxial tensile test. The elastic diagram (stress-strain curve) is built with force and elongation information until the sample rupture.
CASE12accet Specimen 1 Data File.xls	Biomechanical test of left common carotid artery - excel file related to the chart: this excel file contains precisely all the information that enabled the creation of the chart related to this sample. It comprises critical data such as displacement observed during the tensile test (mm), the load applied (N), strain (deformation), tension (N/cm), and stress (N/m2).
CASE12accet.TXT	Biomechanical test of left common carotid artery - biomechanical test report: the biomechanical test device produces a report detailing the experiments' conditions, such as temperature and humidity, as well as the width and thickness of the sample.
CASE12asceCurve Fitted.jpg	Biomechanical test of left subclavian artery – chart represents the elastic diagram related to this uniaxial tensile test. The elastic diagram (stress-strain curve) is built with force and elongation information until the sample rupture.
CASE12ascet Specimen 1 Data File.xls	Biomechanical test of left subclavian artery - excel file related to the chart: this excel file contains precisely all the information that enabled the creation of the chart related to this sample. It comprises critical data such as displacement observed during the tensile test (mm), the load applied (N), strain (deformation), tension (N/cm), and stress (N/m2).
CASE12ascet.TXT	Biomechanical test of left subclavian artery - biomechanical test report: the biomechanical test device produces a report detailing the experiments' conditions, such as temperature and humidity, as well as the width and thickness of the sample.
CASE12tbct Curve Fitted.jpg	Biomechanical test of brachiocephalic trunk – chart represents the elastic diagram related to this uniaxial tensile test. The elastic diagram (stress-strain curve) is built with force and elongation information until the sample rupture.
CASE12tbct Specimen 1 Data File.xls	Biomechanical test of brachiocephalic trunk - excel file related to the chart: this excel file contains precisely all the information that enabled the creation of the chart related to this sample. It comprises critical data such as displacement observed during the tensile test (mm), the load applied (N), strain (deformation), tension (N/cm), and stress (N/m2).
CASE12tbct.TXT	Biomechanical test of brachiocephalic trunk – biomechanical test report: the biomechanical test device produces a report detailing the experiments' conditions, such as temperature and humidity, as well as the width and thickness of the sample.
CASE 13	

CASE13accet Curve Fitted.jpg	Biomechanical test of left common carotid artery – chart represents the elastic diagram related to this uniaxial tensile test. The elastic diagram (stress-strain curve) is built with force and elongation information until the sample rupture.
CASE13accet Specimen 1 Data File.xls	Biomechanical test of left common carotid artery - excel file related to the chart: this excel file contains precisely all the information that enabled the creation of the chart related to this sample. It comprises critical data such as displacement observed during the tensile test (mm), the load applied (N), strain (deformation), tension (N/cm), and stress (N/m2).
CASE13accet.TXT	Biomechanical test of left common carotid artery - biomechanical test report: the biomechanical test device produces a report detailing the experiments' conditions, such as temperature and humidity, as well as the width and thickness of the sample.
CASE13amst Curve Fitted.jpg	Biomechanical test of superior mesenteric artery – chart represents the elastic diagram related to this uniaxial tensile test. The elastic diagram (stress-strain curve) is built with force and elongation information until the sample rupture.
CASE13amst Specimen 1 Data File.xls	Biomechanical test of superior mesenteric artery - excel file related to the chart: this excel file contains precisely all the information that enabled the creation of the chart related to this sample. It comprises critical data such as displacement observed during the tensile test (mm), the load applied (N), strain (deformation), tension (N/cm), and stress (N/m2).
CASE13amst.TXT	Biomechanical test of superior mesenteric artery - biomechanical test report: the biomechanical test device produces a report detailing the experiments' conditions, such as temperature and humidity, as well as the width and thickness of the sample.
CASE13ardt Curve Fitted.jpg	Biomechanical test of right renal artery – chart represents the elastic diagram related to this uniaxial tensile test. The elastic diagram (stress-strain curve) is built with force and elongation information until the sample rupture.
CASE13ardt Specimen 1 Data File.xls	Biomechanical test of right renal artery - excel file related to the chart: this excel file contains precisely all the information that enabled the creation of the chart related to this sample. It comprises critical data such as displacement observed during the tensile test (mm), the load applied (N), strain (deformation), tension (N/cm), and stress (N/m2).
CASE13ardt.TXT	Biomechanical test of right renal artery - biomechanical test report: the biomechanical test device produces a report detailing the experiments' conditions, such as temperature and humidity, as well as the width and thickness of the sample.
CASE13aret Curve Fitted.jpg	Biomechanical test of left renal artery – chart represents the elastic diagram related to this uniaxial tensile test. The elastic diagram (stress-strain curve) is built with force and elongation information until the sample rupture.
CASE13aret Specimen 1 Data File.xls	Biomechanical test of left renal artery - excel file related to the chart: this excel file contains precisely all the information that enabled the creation of the chart related to this sample. It comprises critical data such as displacement observed during the tensile test (mm), the load applied (N), strain (deformation), tension (N/cm), and stress (N/m2).
CASE13aret.TXT	Biomechanical test of left renal artery - biomechanical test report: the biomechanical test device produces a report detailing the experiments' conditions, such as temperature and humidity, as well as the width and thickness of the sample.
CASE13ascet Curve Fitted.jpg	Biomechanical test of left subclavian artery – chart represents the elastic diagram related to this uniaxial tensile test. The elastic diagram (stress-strain curve) is built with force and elongation information until the sample rupture.
CASE13ascet Specimen 1 Data File.xls	Biomechanical test of left subclavian artery - excel file related to the chart: this excel file contains precisely all the information that enabled the creation of the chart related to this sample. It comprises critical data such as displacement observed during the tensile test (mm), the load applied (N), strain (deformation), tension (N/cm), and stress (N/m2).
CASE13ascet.TXT	Biomechanical test of left subclavian artery - biomechanical test report: the biomechanical test device produces a report detailing the experiments' conditions, such as temperature and humidity, as well as the width and thickness of the sample.
CASE13tbct Curve Fitted.jpg	Biomechanical test of brachiocephalic trunk – chart represents the elastic diagram related to this uniaxial tensile test. The elastic diagram (stress-strain curve) is built with force and elongation information until the sample rupture.
CASE13tbct Specimen 1 Data File.xls	Biomechanical test of brachiocephalic trunk - excel file related to the chart: this excel file contains precisely all the information that enabled the creation of the chart related to this sample. It comprises critical data such as displacement observed during the tensile test (mm), the load applied (N), strain (deformation), tension (N/cm), and stress (N/m2).
CASE13tbct.TXT	Biomechanical test of brachiocephalic trunk – biomechanical test report: the biomechanical test device produces a report detailing the experiments' conditions, such as temperature and humidity, as well as the width and thickness of the sample.
CASE 14	

CASE14accet Curve Fitted.jpg	Biomechanical test of left common carotid artery – chart represents the elastic diagram related to this uniaxial tensile test. The elastic diagram (stress-strain curve) is built with force and elongation information until the sample rupture.
CASE14accet Specimen 1 Data File.xls	Biomechanical test of left common carotid artery - excel file related to the chart: this excel file contains precisely all the information that enabled the creation of the chart related to this sample. It comprises critical data such as displacement observed during the tensile test (mm), the load applied (N), strain (deformation), tension (N/cm), and stress (N/m2).
CASE14accet.TXT	Biomechanical test of left common carotid artery - biomechanical test report: the biomechanical test device produces a report detailing the experiments' conditions, such as temperature and humidity, as well as the width and thickness of the sample.
CASE14ardt Curve Fitted.jpg	Biomechanical test of right renal artery – chart represents the elastic diagram related to this uniaxial tensile test. The elastic diagram (stress-strain curve) is built with force and elongation information until the sample rupture.
CASE14ardt Specimen 1 Data File.xls	Biomechanical test of right renal artery - excel file related to the chart: this excel file contains precisely all the information that enabled the creation of the chart related to this sample. It comprises critical data such as displacement observed during the tensile test (mm), the load applied (N), strain (deformation), tension (N/cm), and stress (N/m2).
CASE14ardt.TXT	Biomechanical test of right renal artery - biomechanical test report: the biomechanical test device produces a report detailing the experiments' conditions, such as temperature and humidity, as well as the width and thickness of the sample.
CASE14aret Curve Fitted.jpg	Biomechanical test of left renal artery – chart represents the elastic diagram related to this uniaxial tensile test. The elastic diagram (stress-strain curve) is built with force and elongation information until the sample rupture.
CASE14aret Specimen 1 Data File.xls	Biomechanical test of left renal artery - excel file related to the chart: this excel file contains precisely all the information that enabled the creation of the chart related to this sample. It comprises critical data such as displacement observed during the tensile test (mm), the load applied (N), strain (deformation), tension (N/cm), and stress (N/m2).
CASE14aret.TXT	Biomechanical test of left renal artery - biomechanical test report: the biomechanical test device produces a report detailing the experiments' conditions, such as temperature and humidity, as well as the width and thickness of the sample.
CASE14ascet Curve Fitted.jpg	Biomechanical test of left subclavian artery – chart represents the elastic diagram related to this uniaxial tensile test. The elastic diagram (stress-strain curve) is built with force and elongation information until the sample rupture.
CASE14ascet Specimen 1 Data File.xls	Biomechanical test of left subclavian artery - excel file related to the chart: this excel file contains precisely all the information that enabled the creation of the chart related to this sample. It comprises critical data such as displacement observed during the tensile test (mm), the load applied (N), strain (deformation), tension (N/cm), and stress (N/m2).
CASE14ascet.TXT	Biomechanical test of left subclavian artery - biomechanical test report: the biomechanical test device produces a report detailing the experiments' conditions, such as temperature and humidity, as well as the width and thickness of the sample.
CASE14tbct Curve Fitted.jpg	Biomechanical test of brachiocephalic trunk – chart represents the elastic diagram related to this uniaxial tensile test. The elastic diagram (stress-strain curve) is built with force and elongation information until the sample rupture.
CASE14tbct Specimen 1 Data File.xls	Biomechanical test of brachiocephalic trunk - excel file related to the chart: this excel file contains precisely all the information that enabled the creation of the chart related to this sample. It comprises critical data such as displacement observed during the tensile test (mm), the load applied (N), strain (deformation), tension (N/cm), and stress (N/m2).
CASE14tbct.TXT	Biomechanical test of brachiocephalic trunk – biomechanical test report: the biomechanical test device produces a report detailing the experiments' conditions, such as temperature and humidity, as well as the width and thickness of the sample.
CASE 15	

CASE15 acce Curve Fitted.jpg	Biomechanical test of left common carotid artery – chart represents the elastic diagram related to this uniaxial tensile test. The elastic diagram (stress-strain curve) is built with force and elongation information until the sample rupture.
CASE15 acce Specimen 1 Data File.xls	Biomechanical test of left common carotid artery - excel file related to the chart: this excel file contains precisely all the information that enabled the creation of the chart related to this sample. It comprises critical data such as displacement observed during the tensile test (mm), the load applied (N), strain (deformation), tension (N/cm), and stress (N/m2).
CASE15 acce.TXT	Biomechanical test of left common carotid artery - biomechanical test report: the biomechanical test device produces a report detailing the experiments' conditions, such as temperature and humidity, as well as the width and thickness of the sample.
CASE15 ascet Curve Fitted.jpg	Biomechanical test of left subclavian artery – chart represents the elastic diagram related to this uniaxial tensile test. The elastic diagram (stress-strain curve) is built with force and elongation information until the sample rupture.
CASE15 ascet Specimen 1 Data File.xls	Biomechanical test of left subclavian artery - excel file related to the chart: this excel file contains precisely all the information that enabled the creation of the chart related to this sample. It comprises critical data such as displacement observed during the tensile test (mm), the load applied (N), strain (deformation), tension (N/cm), and stress (N/m2).
CASE15 ascet.TXT	Biomechanical test of left subclavian artery - biomechanical test report: the biomechanical test device produces a report detailing the experiments' conditions, such as temperature and humidity, as well as the width and thickness of the sample.
CASE15 tbct Curve Fitted.jpg	Biomechanical test of brachiocephalic trunk – chart represents the elastic diagram related to this uniaxial tensile test. The elastic diagram (stress-strain curve) is built with force and elongation information until the sample rupture.
CASE15 tbct Specimen 1 Data File.xls	Biomechanical test of brachiocephalic trunk - excel file related to the chart: this excel file contains precisely all the information that enabled the creation of the chart related to this sample. It comprises critical data such as displacement observed during the tensile test (mm), the load applied (N), strain (deformation), tension (N/cm), and stress (N/m2).
CASE15 tbct.TXT	Biomechanical test of brachiocephalic trunk – biomechanical test report: the biomechanical test device produces a report detailing the experiments' conditions, such as temperature and humidity, as well as the width and thickness of the sample.
CASE15 tct Curve Fitted.jpg	Biomechanical test of celiac trunk – chart represents the elastic diagram related to this uniaxial tensile test. The elastic diagram (stress-strain curve) is built with force and elongation information until the sample rupture.
CASE15 tct Specimen 1 Data File.xls	Biomechanical test of celiac trunk - excel file related to the chart: this excel file contains precisely all the information that enabled the creation of the chart related to this sample. It comprises critical data such as displacement observed during the tensile test (mm), the load applied (N), strain (deformation), tension (N/cm), and stress (N/m2).
CASE15 tct.TXT	Biomechanical test of celiac trunk - biomechanical test report: the biomechanical test device produces a report detailing the experiments' conditions, such as temperature and humidity, as well as the width and thickness of the sample.
CASE 16	

CASE16accet Curve Fitted.jpg	Biomechanical test of left common carotid artery – chart represents the elastic diagram related to this uniaxial tensile test. The elastic diagram (stress-strain curve) is built with force and elongation information until the sample rupture.
CASE16accet Specimen 1 Data File.xls	Biomechanical test of left common carotid artery - excel file related to the chart: this excel file contains precisely all the information that enabled the creation of the chart related to this sample. It comprises critical data such as displacement observed during the tensile test (mm), the load applied (N), strain (deformation), tension (N/cm), and stress (N/m2).
CASE16accet.TXT	Biomechanical test of left common carotid artery - biomechanical test report: the biomechanical test device produces a report detailing the experiments' conditions, such as temperature and humidity, as well as the width and thickness of the sample.
CASE16ardt Curve Fitted.jpg	Biomechanical test of right renal artery – chart represents the elastic diagram related to this uniaxial tensile test. The elastic diagram (stress-strain curve) is built with force and elongation information until the sample rupture.
CASE16ardt Specimen 1 Data File.xls	Biomechanical test of right renal artery - excel file related to the chart: this excel file contains precisely all the information that enabled the creation of the chart related to this sample. It comprises critical data such as displacement observed during the tensile test (mm), the load applied (N), strain (deformation), tension (N/cm), and stress (N/m2).
CASE16ardt.TXT	Biomechanical test of right renal artery - biomechanical test report: the biomechanical test device produces a report detailing the experiments' conditions, such as temperature and humidity, as well as the width and thickness of the sample.
CASE16ascet Curve Fitted.jpg	Biomechanical test of left subclavian artery – chart represents the elastic diagram related to this uniaxial tensile test. The elastic diagram (stress-strain curve) is built with force and elongation information until the sample rupture.
CASE16ascet Specimen 1 Data File.xls	Biomechanical test of left subclavian artery - excel file related to the chart: this excel file contains precisely all the information that enabled the creation of the chart related to this sample. It comprises critical data such as displacement observed during the tensile test (mm), the load applied (N), strain (deformation), tension (N/cm), and stress (N/m2).
CASE16ascet.TXT	Biomechanical test of left subclavian artery - biomechanical test report: the biomechanical test device produces a report detailing the experiments' conditions, such as temperature and humidity, as well as the width and thickness of the sample.
CASE16tbct Curve Fitted.jpg	Biomechanical test of brachiocephalic trunk – chart represents the elastic diagram related to this uniaxial tensile test. The elastic diagram (stress-strain curve) is built with force and elongation information until the sample rupture.
CASE16tbct Specimen 1 Data File.xls	Biomechanical test of brachiocephalic trunk - excel file related to the chart: this excel file contains precisely all the information that enabled the creation of the chart related to this sample. It comprises critical data such as displacement observed during the tensile test (mm), the load applied (N), strain (deformation), tension (N/cm), and stress (N/m2).
CASE16tbct.TXT	Biomechanical test of brachiocephalic trunk – biomechanical test report: the biomechanical test device produces a report detailing the experiments' conditions, such as temperature and humidity, as well as the width and thickness of the sample.
CASE 17	

CASE17tct Curve Fitted.jpg	Biomechanical test of celiac trunk – chart represents the elastic diagram related to this uniaxial tensile test. The elastic diagram (stress-strain curve) is built with force and elongation information until the sample rupture.
CASE17tct Specimen 1 Data File.xls	Biomechanical test of celiac trunk - excel file related to the chart: this excel file contains precisely all the information that enabled the creation of the chart related to this sample. It comprises critical data such as displacement observed during the tensile test (mm), the load applied (N), strain (deformation), tension (N/cm), and stress (N/m2).
CASE17tct.TXT	Biomechanical test of celiac trunk - biomechanical test report: the biomechanical test device produces a report detailing the experiments' conditions, such as temperature and humidity, as well as the width and thickness of the sample.
CASE 18	

CASE18ascet Curve Fitted.jpg	Biomechanical test of left subclavian artery – chart represents the elastic diagram related to this uniaxial tensile test. The elastic diagram (stress-strain curve) is built with force and elongation information until the sample rupture.
CASE18ascet Specimen 1 Data File.xls	Biomechanical test of left subclavian artery - excel file related to the chart: this excel file contains precisely all the information that enabled the creation of the chart related to this sample. It comprises critical data such as displacement observed during the tensile test (mm), the load applied (N), strain (deformation), tension (N/cm), and stress (N/m2).
CASE18ascet.TXT	Biomechanical test of left subclavian artery - biomechanical test report: the biomechanical test device produces a report detailing the experiments' conditions, such as temperature and humidity, as well as the width and thickness of the sample.
CASE18tbct Curve Fitted.jpg	Biomechanical test of brachiocephalic trunk – chart represents the elastic diagram related to this uniaxial tensile test. The elastic diagram (stress-strain curve) is built with force and elongation information until the sample rupture.
CASE18tbct Specimen 1 Data File.xls	Biomechanical test of brachiocephalic trunk - excel file related to the chart: this excel file contains precisely all the information that enabled the creation of the chart related to this sample. It comprises critical data such as displacement observed during the tensile test (mm), the load applied (N), strain (deformation), tension (N/cm), and stress (N/m2).
CASE18tbct.TXT	Biomechanical test of brachiocephalic trunk – biomechanical test report: the biomechanical test device produces a report detailing the experiments' conditions, such as temperature and humidity, as well as the width and thickness of the sample.
CASE 19	

CASE19accet Curve Fitted.jpg	Biomechanical test of left common carotid artery – chart represents the elastic diagram related to this uniaxial tensile test. The elastic diagram (stress-strain curve) is built with force and elongation information until the sample rupture.
CASE19accet Specimen 1 Data File.xls	Biomechanical test of left common carotid artery - excel file related to the chart: this excel file contains precisely all the information that enabled the creation of the chart related to this sample. It comprises critical data such as displacement observed during the tensile test (mm), the load applied (N), strain (deformation), tension (N/cm), and stress (N/m2).
CASE19accet.TXT	Biomechanical test of left common carotid artery - biomechanical test report: the biomechanical test device produces a report detailing the experiments' conditions, such as temperature and humidity, as well as the width and thickness of the sample.
CASE19tbct Curve Fitted.jpg	Biomechanical test of brachiocephalic trunk – chart represents the elastic diagram related to this uniaxial tensile test. The elastic diagram (stress-strain curve) is built with force and elongation information until the sample rupture.
CASE19tbct Specimen 1 Data File.xls	Biomechanical test of brachiocephalic trunk - excel file related to the chart: this excel file contains precisely all the information that enabled the creation of the chart related to this sample. It comprises critical data such as displacement observed during the tensile test (mm), the load applied (N), strain (deformation), tension (N/cm), and stress (N/m2).
CASE19tbct.TXT	Biomechanical test of brachiocephalic trunk – biomechanical test report: the biomechanical test device produces a report detailing the experiments' conditions, such as temperature and humidity, as well as the width and thickness of the sample.
CASE 20	

CASE20amst Curve Fitted.jpg	Biomechanical test of superior mesenteric artery – chart represents the elastic diagram related to this uniaxial tensile test. The elastic diagram (stress-strain curve) is built with force and elongation information until the sample rupture.
CASE20amst Data File.xls	Biomechanical test of superior mesenteric artery - excel file related to the chart: this excel file contains precisely all the information that enabled the creation of the chart related to this sample. It comprises critical data such as displacement observed during the tensile test (mm), the load applied (N), strain (deformation), tension (N/cm), and stress (N/m2).
CASE20amst Data report.txt	Biomechanical test of superior mesenteric artery - biomechanical test report: the biomechanical test device produces a report detailing the experiments' conditions, such as temperature and humidity, as well as the width and thickness of the sample.
CASE20ardt Curve Fitted.jpg	Biomechanical test of right renal artery – chart represents the elastic diagram related to this uniaxial tensile test. The elastic diagram (stress-strain curve) is built with force and elongation information until the sample rupture.
CASE20ardt Specimen 1 Data File.xls	Biomechanical test of right renal artery - excel file related to the chart: this excel file contains precisely all the information that enabled the creation of the chart related to this sample. It comprises critical data such as displacement observed during the tensile test (mm), the load applied (N), strain (deformation), tension (N/cm), and stress (N/m2).
CASE20ardt.TXT	Biomechanical test of right renal artery - biomechanical test report: the biomechanical test device produces a report detailing the experiments' conditions, such as temperature and humidity, as well as the width and thickness of the sample.
CASE20aret Curve Fitted.jpg	Biomechanical test of left renal artery – chart represents the elastic diagram related to this uniaxial tensile test. The elastic diagram (stress-strain curve) is built with force and elongation information until the sample rupture.
CASE20aret Specimen 1 Data File.xls	Biomechanical test of left renal artery - excel file related to the chart: this excel file contains precisely all the information that enabled the creation of the chart related to this sample. It comprises critical data such as displacement observed during the tensile test (mm), the load applied (N), strain (deformation), tension (N/cm), and stress (N/m2).
CASE20aret.TXT	Biomechanical test of left renal artery - biomechanical test report: the biomechanical test device produces a report detailing the experiments' conditions, such as temperature and humidity, as well as the width and thickness of the sample.
CASE20tct Curve Fitted.jpg	Biomechanical test of celiac trunk – chart represents the elastic diagram related to this uniaxial tensile test. The elastic diagram (stress-strain curve) is built with force and elongation information until the sample rupture.
CASE20tct Specimen 1 Data File.xls	Biomechanical test of celiac trunk - excel file related to the chart: this excel file contains precisely all the information that enabled the creation of the chart related to this sample. It comprises critical data such as displacement observed during the tensile test (mm), the load applied (N), strain (deformation), tension (N/cm), and stress (N/m2).
CASE20tct.TXT	Biomechanical test of celiac trunk - biomechanical test report: the biomechanical test device produces a report detailing the experiments' conditions, such as temperature and humidity, as well as the width and thickness of the sample.
CASE 21	

CASE21accet Curve Fitted.jpg	Biomechanical test of left common carotid artery – chart represents the elastic diagram related to this uniaxial tensile test. The elastic diagram (stress-strain curve) is built with force and elongation information until the sample rupture.
CASE21accet Specimen 1 Data File.xls	Biomechanical test of left common carotid artery - excel file related to the chart: this excel file contains precisely all the information that enabled the creation of the chart related to this sample. It comprises critical data such as displacement observed during the tensile test (mm), the load applied (N), strain (deformation), tension (N/cm), and stress (N/m2).
CASE21accet.TXT	Biomechanical test of left common carotid artery - biomechanical test report: the biomechanical test device produces a report detailing the experiments' conditions, such as temperature and humidity, as well as the width and thickness of the sample.
CASE21amst Curve Fitted.jpg	Biomechanical test of superior mesenteric artery – chart represents the elastic diagram related to this uniaxial tensile test. The elastic diagram (stress-strain curve) is built with force and elongation information until the sample rupture.
CASE21amst Specimen 1 Data File.xls	Biomechanical test of superior mesenteric artery - excel file related to the chart: this excel file contains precisely all the information that enabled the creation of the chart related to this sample. It comprises critical data such as displacement observed during the tensile test (mm), the load applied (N), strain (deformation), tension (N/cm), and stress (N/m2).
CASE21amst.TXT	Biomechanical test of superior mesenteric artery - biomechanical test report: the biomechanical test device produces a report detailing the experiments' conditions, such as temperature and humidity, as well as the width and thickness of the sample.
CASE21ascet Curve Fitted.jpg	Biomechanical test of left subclavian artery – chart represents the elastic diagram related to this uniaxial tensile test. The elastic diagram (stress-strain curve) is built with force and elongation information until the sample rupture.
CASE21ascet Specimen 1 Data File.xls	Biomechanical test of left subclavian artery - excel file related to the chart: this excel file contains precisely all the information that enabled the creation of the chart related to this sample. It comprises critical data such as displacement observed during the tensile test (mm), the load applied (N), strain (deformation), tension (N/cm), and stress (N/m2).
CASE21ascet.TXT	Biomechanical test of left subclavian artery - biomechanical test report: the biomechanical test device produces a report detailing the experiments' conditions, such as temperature and humidity, as well as the width and thickness of the sample.
CASE21tbct Curve Fitted.jpg	Biomechanical test of brachiocephalic trunk – chart represents the elastic diagram related to this uniaxial tensile test. The elastic diagram (stress-strain curve) is built with force and elongation information until the sample rupture.
CASE21tbct Specimen 1 Data File.xls	Biomechanical test of brachiocephalic trunk - excel file related to the chart: this excel file contains precisely all the information that enabled the creation of the chart related to this sample. It comprises critical data such as displacement observed during the tensile test (mm), the load applied (N), strain (deformation), tension (N/cm), and stress (N/m2).
CASE21tbct.TXT	Biomechanical test of brachiocephalic trunk – biomechanical test report: the biomechanical test device produces a report detailing the experiments' conditions, such as temperature and humidity, as well as the width and thickness of the sample.
CASE21tct Curve Fitted.jpg	Biomechanical test of celiac trunk – chart represents the elastic diagram related to this uniaxial tensile test. The elastic diagram (stress-strain curve) is built with force and elongation information until the sample rupture.
CASE21tct Specimen 1 Data File.xls	Biomechanical test of celiac trunk - excel file related to the chart: this excel file contains precisely all the information that enabled the creation of the chart related to this sample. It comprises critical data such as displacement observed during the tensile test (mm), the load applied (N), strain (deformation), tension (N/cm), and stress (N/m2).-
CASE21tct.TXT	Biomechanical test of celiac trunk - biomechanical test report: the biomechanical test device produces a report detailing the experiments' conditions, such as temperature and humidity, as well as the width and thickness of the sample.
CASE 22	

CASE22accet Curve Fitted.jpg	Biomechanical test of left common carotid artery – chart represents the elastic diagram related to this uniaxial tensile test. The elastic diagram (stress-strain curve) is built with force and elongation information until the sample rupture.
CASE22accet Specimen 1 Data File.xls	Biomechanical test of left common carotid artery - excel file related to the chart: this excel file contains precisely all the information that enabled the creation of the chart related to this sample. It comprises critical data such as displacement observed during the tensile test (mm), the load applied (N), strain (deformation), tension (N/cm), and stress (N/m2).
CASE22accet Specimen 1 Data report.txt	Biomechanical test of left common carotid artery - biomechanical test report: the biomechanical test device produces a report detailing the experiments' conditions, such as temperature and humidity, as well as the width and thickness of the sample.
CASE22amst Curve Fitted.jpg	Biomechanical test of superior mesenteric artery – chart represents the elastic diagram related to this uniaxial tensile test. The elastic diagram (stress-strain curve) is built with force and elongation information until the sample rupture.
CASE22amst Specimen 1 Data File.xls	Biomechanical test of superior mesenteric artery - excel file related to the chart: this excel file contains precisely all the information that enabled the creation of the chart related to this sample. It comprises critical data such as displacement observed during the tensile test (mm), the load applied (N), strain (deformation), tension (N/cm), and stress (N/m2).
CASE22amst.TXT	Biomechanical test of superior mesenteric artery - biomechanical test report: the biomechanical test device produces a report detailing the experiments' conditions, such as temperature and humidity, as well as the width and thickness of the sample.
CASE22ardt Curve Fitted.jpg	Biomechanical test of right renal artery – chart represents the elastic diagram related to this uniaxial tensile test. The elastic diagram (stress-strain curve) is built with force and elongation information until the sample rupture.
CASE22ardt Specimen 1 Data File.xls	Biomechanical test of right renal artery - excel file related to the chart: this excel file contains precisely all the information that enabled the creation of the chart related to this sample. It comprises critical data such as displacement observed during the tensile test (mm), the load applied (N), strain (deformation), tension (N/cm), and stress (N/m2).
CASE22ardt.TXT	Biomechanical test of right renal artery - biomechanical test report: the biomechanical test device produces a report detailing the experiments' conditions, such as temperature and humidity, as well as the width and thickness of the sample.
CASE22ascet Curve Fitted.jpg	Biomechanical test of left subclavian artery – chart represents the elastic diagram related to this uniaxial tensile test. The elastic diagram (stress-strain curve) is built with force and elongation information until the sample rupture.
CASE22ascet Specimen 1 Data File.xls	Biomechanical test of left subclavian artery - excel file related to the chart: this excel file contains precisely all the information that enabled the creation of the chart related to this sample. It comprises critical data such as displacement observed during the tensile test (mm), the load applied (N), strain (deformation), tension (N/cm), and stress (N/m2).
CASE22ascet.TXT	Biomechanical test of left subclavian artery - biomechanical test report: the biomechanical test device produces a report detailing the experiments' conditions, such as temperature and humidity, as well as the width and thickness of the sample.
CASE22tbct Curve Fitted.jpg	Biomechanical test of brachiocephalic trunk – chart represents the elastic diagram related to this uniaxial tensile test. The elastic diagram (stress-strain curve) is built with force and elongation information until the sample rupture.
CASE22tbct Specimen 1 Data File.xls	Biomechanical test of brachiocephalic trunk - excel file related to the chart: this excel file contains precisely all the information that enabled the creation of the chart related to this sample. It comprises critical data such as displacement observed during the tensile test (mm), the load applied (N), strain (deformation), tension (N/cm), and stress (N/m2).
CASE22tbct.TXT	Biomechanical test of brachiocephalic trunk – biomechanical test report: the biomechanical test device produces a report detailing the experiments' conditions, such as temperature and humidity, as well as the width and thickness of the sample.
CASE 23	

CASE23accet Curve Fitted.jpg	Biomechanical test of left common carotid artery – chart represents the elastic diagram related to this uniaxial tensile test. The elastic diagram (stress-strain curve) is built with force and elongation information until the sample rupture.
CASE23accet Specimen 1 Data File.xls	Biomechanical test of left common carotid artery - excel file related to the chart: this excel file contains precisely all the information that enabled the creation of the chart related to this sample. It comprises critical data such as displacement observed during the tensile test (mm), the load applied (N), strain (deformation), tension (N/cm), and stress (N/m2).
CASE23accet.TXT	Biomechanical test of left common carotid artery - biomechanical test report: the biomechanical test device produces a report detailing the experiments' conditions, such as temperature and humidity, as well as the width and thickness of the sample.
CASE23amst Curve Fitted.jpg	Biomechanical test of superior mesenteric artery – chart represents the elastic diagram related to this uniaxial tensile test. The elastic diagram (stress-strain curve) is built with force and elongation information until the sample rupture.
CASE23amst Specimen 1 Data File.xls	Biomechanical test of superior mesenteric artery - excel file related to the chart: this excel file contains precisely all the information that enabled the creation of the chart related to this sample. It comprises critical data such as displacement observed during the tensile test (mm), the load applied (N), strain (deformation), tension (N/cm), and stress (N/m2).
CASE23amst.TXT	Biomechanical test of superior mesenteric artery - biomechanical test report: the biomechanical test device produces a report detailing the experiments' conditions, such as temperature and humidity, as well as the width and thickness of the sample.
CASE23tbct Curve Fitted.jpg	Biomechanical test of brachiocephalic trunk – chart represents the elastic diagram related to this uniaxial tensile test. The elastic diagram (stress-strain curve) is built with force and elongation information until the sample rupture.
CASE23tbct Specimen 1 Data File.xls	Biomechanical test of brachiocephalic trunk - excel file related to the chart: this excel file contains precisely all the information that enabled the creation of the chart related to this sample. It comprises critical data such as displacement observed during the tensile test (mm), the load applied (N), strain (deformation), tension (N/cm), and stress (N/m2).
CASE23tbct.TXT	Biomechanical test of brachiocephalic trunk – biomechanical test report: the biomechanical test device produces a report detailing the experiments' conditions, such as temperature and humidity, as well as the width and thickness of the sample.
CASE23tct Curve Fitted.jpg	Biomechanical test of celiac trunk – chart represents the elastic diagram related to this uniaxial tensile test. The elastic diagram (stress-strain curve) is built with force and elongation information until the sample rupture.
CASE23tct Specimen 1 Data File.xls	Biomechanical test of celiac trunk - excel file related to the chart: this excel file contains precisely all the information that enabled the creation of the chart related to this sample. It comprises critical data such as displacement observed during the tensile test (mm), the load applied (N), strain (deformation), tension (N/cm), and stress (N/m2).
CASE23tct.TXT	Biomechanical test of celiac trunk - biomechanical test report: the biomechanical test device produces a report detailing the experiments' conditions, such as temperature and humidity, as well as the width and thickness of the sample.
CASE 24	

CASE24accet Curve Fitted.jpg	Biomechanical test of left common carotid artery – chart represents the elastic diagram related to this uniaxial tensile test. The elastic diagram (stress-strain curve) is built with force and elongation information until the sample rupture.
CASE24accet Specimen 1 Data File.xls	Biomechanical test of left common carotid artery - excel file related to the chart: this excel file contains precisely all the information that enabled the creation of the chart related to this sample. It comprises critical data such as displacement observed during the tensile test (mm), the load applied (N), strain (deformation), tension (N/cm), and stress (N/m2).
CASE24accet.TXT	Biomechanical test of left common carotid artery – biomechanical test report: the biomechanical test device produces a report detailing the experiments' conditions, such as temperature and humidity, as well as the width and thickness of the sample.
CASE24 amst.TXT	Biomechanical test of superior mesenteric artery – biomechanical test report: the biomechanical test device produces a report detailing the experiments' conditions, such as temperature and humidity, as well as the width and thickness of the sample.
CASE24amst Curve Fitted.jpg	Biomechanical test of superior mesenteric artery – chart represents the elastic diagram related to this uniaxial tensile test. The elastic diagram (stress-strain curve) is built with force and elongation information until the sample rupture.
CASE24amst Specimen 1 Data File.xls	Biomechanical test of superior mesenteric artery - excel file related to the chart: this excel file contains precisely all the information that enabled the creation of the chart related to this sample. It comprises critical data such as displacement observed during the tensile test (mm), the load applied (N), strain (deformation), tension (N/cm), and stress (N/m2).
CASE24ascet Curve Fitted.jpg	Biomechanical test of left subclavian artery – chart represents the elastic diagram related to this uniaxial tensile test. The elastic diagram (stress-strain curve) is built with force and elongation information until the sample rupture.
CASE24ascet Specimen 1 Data File.xls	Biomechanical test of left subclavian artery - excel file related to the chart: this excel file contains precisely all the information that enabled the creation of the chart related to this sample. It comprises critical data such as displacement observed during the tensile test (mm), the load applied (N), strain (deformation), tension (N/cm), and stress (N/m2).
CASE24ascet.TXT	Biomechanical test of left subclavian artery - biomechanical test report: the biomechanical test device produces a report detailing the experiments' conditions, such as temperature and humidity, as well as the width and thickness of the sample.
CASE24tbct Curve Fitted.jpg	Biomechanical test of brachiocephalic trunk – chart represents the elastic diagram related to this uniaxial tensile test. The elastic diagram (stress-strain curve) is built with force and elongation information until the sample rupture.
CASE24tbct Specimen 1 Data File.xls	Biomechanical test of brachiocephalic trunk - excel file related to the chart: this excel file contains precisely all the information that enabled the creation of the chart related to this sample. It comprises critical data such as displacement observed during the tensile test (mm), the load applied (N), strain (deformation), tension (N/cm), and stress (N/m2).
CASE24tbct.TXT	Biomechanical test of brachiocephalic trunk – biomechanical test report: the biomechanical test device produces a report detailing the experiments' conditions, such as temperature and humidity, as well as the width and thickness of the sample.
CASE 25	

CASE25accet Curve Fitted.jpg	Biomechanical test of left common carotid artery – chart represents the elastic diagram related to this uniaxial tensile test. The elastic diagram (stress-strain curve) is built with force and elongation information until the sample rupture.
CASE25accet Specimen 1 Data File.xls	Biomechanical test of left common carotid artery - excel file related to the chart: this excel file contains precisely all the information that enabled the creation of the chart related to this sample. It comprises critical data such as displacement observed during the tensile test (mm), the load applied (N), strain (deformation), tension (N/cm), and stress (N/m2).
CASE25accet.TXT	Biomechanical test of left common carotid artery - biomechanical test report: the biomechanical test device produces a report detailing the experiments' conditions, such as temperature and humidity, as well as the width and thickness of the sample.
CASE25amst Curve Fitted.jpg	Biomechanical test of superior mesenteric artery – chart represents the elastic diagram related to this uniaxial tensile test. The elastic diagram (stress-strain curve) is built with force and elongation information until the sample rupture.
CASE25ams.xls	Biomechanical test of superior mesenteric artery - excel file related to the chart: this excel file contains precisely all the information that enabled the creation of the chart related to this sample. It comprises critical data such as displacement observed during the tensile test (mm), the load applied (N), strain (deformation), tension (N/cm), and stress (N/m2).
CASE25amst.TXT	Biomechanical test of superior mesenteric artery - biomechanical test report: the biomechanical test device produces a report detailing the experiments' conditions, such as temperature and humidity, as well as the width and thickness of the sample.
CASE25ardt Curve Fitted.jpg	Biomechanical test of right renal artery – chart represents the elastic diagram related to this uniaxial tensile test. The elastic diagram (stress-strain curve) is built with force and elongation information until the sample rupture.
CASE25ardt Specimen 1 Data File.xls	Biomechanical test of right renal artery - excel file related to the chart: this excel file contains precisely all the information that enabled the creation of the chart related to this sample. It comprises critical data such as displacement observed during the tensile test (mm), the load applied (N), strain (deformation), tension (N/cm), and stress (N/m2).
CASE25ardt.TXT	Biomechanical test of right renal artery - biomechanical test report: the biomechanical test device produces a report detailing the experiments' conditions, such as temperature and humidity, as well as the width and thickness of the sample.
CASE25ascet Curve Fitted.jpg	Biomechanical test of left subclavian artery – chart represents the elastic diagram related to this uniaxial tensile test. The elastic diagram (stress-strain curve) is built with force and elongation information until the sample rupture.
CASE25ascet Specimen 1 Data File.xls	Biomechanical test of left subclavian artery - excel file related to the chart: this excel file contains precisely all the information that enabled the creation of the chart related to this sample. It comprises critical data such as displacement observed during the tensile test (mm), the load applied (N), strain (deformation), tension (N/cm), and stress (N/m2).
CASE25ascet.TXT	Biomechanical test of left subclavian artery - biomechanical test report: the biomechanical test device produces a report detailing the experiments' conditions, such as temperature and humidity, as well as the width and thickness of the sample.
CASE25tbct Curve Fitted.jpg	Biomechanical test of brachiocephalic trunk – chart represents the elastic diagram related to this uniaxial tensile test. The elastic diagram (stress-strain curve) is built with force and elongation information until the sample rupture.
CASE25tbct Specimen 1 Data File.xls	Biomechanical test of brachiocephalic trunk - excel file related to the chart: this excel file contains precisely all the information that enabled the creation of the chart related to this sample. It comprises critical data such as displacement observed during the tensile test (mm), the load applied (N), strain (deformation), tension (N/cm), and stress (N/m2).
CASE25tbct.TXT	Biomechanical test of brachiocephalic trunk – biomechanical test report: the biomechanical test device produces a report detailing the experiments' conditions, such as temperature and humidity, as well as the width and thickness of the sample.
CASE25tct Curve Fitted.jpg	Biomechanical test of celiac trunk – chart represents the elastic diagram related to this uniaxial tensile test. The elastic diagram (stress-strain curve) is built with force and elongation information until the sample rupture.
CASE25tct Specimen 1 Data File.xls	Biomechanical test of celiac trunk - excel file related to the chart: this excel file contains precisely all the information that enabled the creation of the chart related to this sample. It comprises critical data such as displacement observed during the tensile test (mm), the load applied (N), strain (deformation), tension (N/cm), and stress (N/m2).
CASE25tct.TXT	Biomechanical test of celiac trunk - biomechanical test report: the biomechanical test device produces a report detailing the experiments' conditions, such as temperature and humidity, as well as the width and thickness of the sample.
CASE 26	

CASE26accet Curve Fitted.jpg	Biomechanical test of left common carotid artery – chart represents the elastic diagram related to this uniaxial tensile test. The elastic diagram (stress-strain curve) is built with force and elongation information until the sample rupture.
CASE26accet Specimen 1 Data File.xls	Biomechanical test of left common carotid artery - excel file related to the chart: this excel file contains precisely all the information that enabled the creation of the chart related to this sample. It comprises critical data such as displacement observed during the tensile test (mm), the load applied (N), strain (deformation), tension (N/cm), and stress (N/m2).
CASE26accet.TXT	Biomechanical test of left common carotid artery - biomechanical test report: the biomechanical test device produces a report detailing the experiments' conditions, such as temperature and humidity, as well as the width and thickness of the sample.
CASE26ardt Curve Fitted.jpg	Biomechanical test of right renal artery – chart represents the elastic diagram related to this uniaxial tensile test. The elastic diagram (stress-strain curve) is built with force and elongation information until the sample rupture.
CASE26ardt Specimen 1 Data File.xls	Biomechanical test of right renal artery - excel file related to the chart: this excel file contains precisely all the information that enabled the creation of the chart related to this sample. It comprises critical data such as displacement observed during the tensile test (mm), the load applied (N), strain (deformation), tension (N/cm), and stress (N/m2).
CASE26ardt.TXT	Biomechanical test of right renal artery - biomechanical test report: the biomechanical test device produces a report detailing the experiments' conditions, such as temperature and humidity, as well as the width and thickness of the sample.
CASE26ascet Curve Fitted.jpg	Biomechanical test of left subclavian artery – chart represents the elastic diagram related to this uniaxial tensile test. The elastic diagram (stress-strain curve) is built with force and elongation information until the sample rupture.
CASE26ascet Specimen 1 Data File.xls	Biomechanical test of left subclavian artery - excel file related to the chart: this excel file contains precisely all the information that enabled the creation of the chart related to this sample. It comprises critical data such as displacement observed during the tensile test (mm), the load applied (N), strain (deformation), tension (N/cm), and stress (N/m2).
CASE26ascet.TXT	Biomechanical test of left subclavian artery - biomechanical test report: the biomechanical test device produces a report detailing the experiments' conditions, such as temperature and humidity, as well as the width and thickness of the sample.
CASE26tbct Curve Fitted.jpg	Biomechanical test of brachiocephalic trunk – chart represents the elastic diagram related to this uniaxial tensile test. The elastic diagram (stress-strain curve) is built with force and elongation information until the sample rupture.
CASE26tbct Specimen 1 Data File.xls	Biomechanical test of brachiocephalic trunk - excel file related to the chart: this excel file contains precisely all the information that enabled the creation of the chart related to this sample. It comprises critical data such as displacement observed during the tensile test (mm), the load applied (N), strain (deformation), tension (N/cm), and stress (N/m2).
CASE26tbct.TXT	Biomechanical test of brachiocephalic trunk – biomechanical test report: the biomechanical test device produces a report detailing the experiments' conditions, such as temperature and humidity, as well as the width and thickness of the sample.
CASE 27	

CASE27accet Curve Fitted.jpg	Biomechanical test of left common carotid artery – chart represents the elastic diagram related to this uniaxial tensile test. The elastic diagram (stress-strain curve) is built with force and elongation information until the sample rupture.
CASE27accet Specimen 1 Data File.xls	Biomechanical test of left common carotid artery - excel file related to the chart: this excel file contains precisely all the information that enabled the creation of the chart related to this sample. It comprises critical data such as displacement observed during the tensile test (mm), the load applied (N), strain (deformation), tension (N/cm), and stress (N/m2).
CASE27accet.TXT	Biomechanical test of left common carotid artery - biomechanical test report: the biomechanical test device produces a report detailing the experiments' conditions, such as temperature and humidity, as well as the width and thickness of the sample.
CASE27amst Curve Fitted.jpg	Biomechanical test of superior mesenteric artery – chart represents the elastic diagram related to this uniaxial tensile test. The elastic diagram (stress-strain curve) is built with force and elongation information until the sample rupture.
CASE27amst Specimen 1 Data File.xls	Biomechanical test of superior mesenteric artery - excel file related to the chart: this excel file contains precisely all the information that enabled the creation of the chart related to this sample. It comprises critical data such as displacement observed during the tensile test (mm), the load applied (N), strain (deformation), tension (N/cm), and stress (N/m2).
CASE27amst.TXT	Biomechanical test of superior mesenteric artery - biomechanical test report: the biomechanical test device produces a report detailing the experiments' conditions, such as temperature and humidity, as well as the width and thickness of the sample.
CASE27ardt Curve Fitted.jpg	Biomechanical test of right renal artery – chart represents the elastic diagram related to this uniaxial tensile test. The elastic diagram (stress-strain curve) is built with force and elongation information until the sample rupture.
CASE27ardt Specimen 1 Data File.xls	Biomechanical test of right renal artery - excel file related to the chart: this excel file contains precisely all the information that enabled the creation of the chart related to this sample. It comprises critical data such as displacement observed during the tensile test (mm), the load applied (N), strain (deformation), tension (N/cm), and stress (N/m2).
CASE27ardt.TXT	Biomechanical test of right renal artery - biomechanical test report: the biomechanical test device produces a report detailing the experiments' conditions, such as temperature and humidity, as well as the width and thickness of the sample.
CASE27ascet Curve Fitted.jpg	Biomechanical test of left subclavian artery – chart represents the elastic diagram related to this uniaxial tensile test. The elastic diagram (stress-strain curve) is built with force and elongation information until the sample rupture.
CASE27ascet Specimen 1 Data File.xls	Biomechanical test of left subclavian artery - excel file related to the chart: this excel file contains precisely all the information that enabled the creation of the chart related to this sample. It comprises critical data such as displacement observed during the tensile test (mm), the load applied (N), strain (deformation), tension (N/cm), and stress (N/m2).
CASE27ascet.TXT	Biomechanical test of left subclavian artery - biomechanical test report: the biomechanical test device produces a report detailing the experiments' conditions, such as temperature and humidity, as well as the width and thickness of the sample.
CASE27tbct Curve Fitted.jpg	Biomechanical test of brachiocephalic trunk – chart represents the elastic diagram related to this uniaxial tensile test. The elastic diagram (stress-strain curve) is built with force and elongation information until the sample rupture.
CASE27tbct Specimen 1 Data File.xls	Biomechanical test of brachiocephalic trunk - excel file related to the chart: this excel file contains precisely all the information that enabled the creation of the chart related to this sample. It comprises critical data such as displacement observed during the tensile test (mm), the load applied (N), strain (deformation), tension (N/cm), and stress (N/m2).
CASE27tbct.TXT	Biomechanical test of brachiocephalic trunk – biomechanical test report: the biomechanical test device produces a report detailing the experiments' conditions, such as temperature and humidity, as well as the width and thickness of the sample.
General File	

Demographic Data Table.xls	Table containing age and gender of all donors

## 2 Experimental Design, Materials and Methods

### 2.1 * biomechanical uniaxial test

The aortic branches submitted to biomechanical analysis were: brachiocephalic trunk, left carotid artery, left subclavian artery, celiac trunk, superior mesenteric artery, and renal arteries. During the autopsy procedure, the whole aorta (thoracic and abdominal parts, up to the iliac vessels) was harvested along with its branches. They were then frozen and stored for the shorter period possible, as the autopsy procedure could happen when there were not available collaborators to run the experiment. Stemper et al. [3] experimentally observed no significant difference in biomechanical behavior between fresh vessel samples and frozen specimens up to 90 days. At the date assigned for the experiment, each aorta was carefully defrosted at room temperature, dissected, and samples were collected from its branches. The aortas were directed to a different research protocol.

The collected arterial segments were kept in a 0.9% saline solution chilled at 4 °C until the biomechanical test was executed within the maximum period of 48 hrs after dissection. The arteries were longitudinally opened and sectioned in strips 4 mm wide for the biomechanical tensile test. Then, the fragments were attached to a clamp system connected to the INSTRON SPEC 2200 device, responsible for pulling the fragments during the uniaxial tensile test. The test was coordinated using INSPEC software. Its standardization was established for aorta samples by Raghavan et al. [4], and was largely reproduced in posterior works [5,6]. The association between deformation values and respective strength values (stress) is made through the PC throughout the tensile test, using the software SERIES IX. A graph denominated elastic diagram is generated.

The uniaxial biomechanical properties measured were: failure stress, failure tension, and failure strain.

## Ethics Statement

The dataset was approved by the institutional review board (Ethical Committee of University of São Paulo School of Medicine #263/15 and #0027/17). All specimens were harvested during autopsy procedure in the Service for verification of death of University of São Paulo (SVOC – USP). Family members of the donors sign an informed consent applied by the SVOC – USP.

## Declaration of Competing Interest

The authors declare that they have no known competing financial interests or personal relationships which have, or could be perceived to have, influenced the work reported in this article.
